# Editorial: Footprints of Immune Cells in the Type 1 Diabetic Pancreas

**DOI:** 10.3389/fendo.2021.767012

**Published:** 2021-10-14

**Authors:** Todd M. Brusko, Roberto Mallone, Teresa Rodriguez-Calvo

**Affiliations:** ^1^ Department of Pathology, Immunology and Laboratory Medicine, University of Florida, Gainesville, FL, United States; ^2^ Department of Pediatrics, College of Medicine, University of Florida, Gainesville, FL, United States; ^3^ Diabetes Institute, University of Florida, Gainesville, FL, United States; ^4^ Université de Paris, Institut Cochin, CNRS, INSERM, Paris, France; ^5^ Assistance Publique Hôpitaux de Paris, Cochin Hospital, Service de Diabétologie et Immunologie Clinique, Paris, France; ^6^ Institute of Diabetes Research, Helmholtz Zentrum München, German Research Center for Environmental Health, Munich-Neuherberg, Germany; ^7^ German Center for Diabetes Research (DZD), Neuherberg, Germany

**Keywords:** type 1 diabetes, immune cells, pancreas, beta cells, autoimmune disease

*The Footprints of Immune Cells in the Type 1 Diabetic Pancreas* Research Topic represents a collection of review articles, perspective pieces, and original research articles, which together create a conceptual framework describing the interactions between cells within the islets of Langerhans and components of the innate and adaptive immune system. The cellular interactions occurring within tissues represent crucial events during the natural history and pathogenesis of the disease, and are not completely understood when studied in isolation. Access to human pancreas samples, through a number of tissue biorepositories, has dramatically improved our collective understanding of type 1 diabetes (T1D), highlighting a number of outstanding questions and key knowledge gaps within the field. Although T1D has been traditionally considered a disease of autoimmune origin, the concept of intracellular stress as a triggering event has gained considerable attention as a means by which β-cells may contribute to their own demise.

In a mini-review article, Piganelli et al. described how, under conditions of high endoplasmic reticulum (ER) stress, β-cells are prone to changes in function and immunogenicity that could lead to the formation of novel antigenic epitopes. The authors described how the unique physiology of β-cells and the extreme metabolic burden of insulin synthesis and secretion, may make them more vulnerable to certain environmental stressors. In addition, genetic risk variants expressed within β-cells may predispose susceptible individuals to increased stress and damage.

Along these lines, and with special relevance given the global coronavirus disease 2019 (COVID-19) pandemic, Fignani et al. investigated the expression of the severe acute respiratory syndrome- coronavirus 2 (SARS-CoV-2) receptor Angiotensin I-Converting Enzyme Type 2 (ACE2) in β-cells and within the islet microvasculature. The authors examined human pancreas samples and reported that ACE2 could be detected in human islets. It was preferentially expressed in distinct subsets of β-cells, in addition to pericytes and some ductal cells, though β-cells primarily expressed the short-ACE2 isoform, which lacks the SARS-CoV-2 high-affinity binding sites. Proinflammatory cytokines increased the expression of ACE2 in a β-cell line and in isolated islets, indicating a potential link between inflammation and ACE2 expression. These data add an additional element to a timely question related to whether or not SARS-CoV-2 can actively infect β-cells and contribute to diabetes.

In a systematic review article, Colli et al. evaluated selected publicly available RNA-seq datasets of pancreatic human islets or FACS-purified human β-cells exposed to: 1) pro-inflammatory stimuli (IL-1β + IFN-γ or IFN-α), 2) metabolic stressors (palmitate), or 3) the local environment present during T1D development (primary β-cells from patients). The biological processes regulated by the transcripts from β-cells exposed to IFN-α closely recapitulated those observed in β-cells from T1D subjects. Transcriptional profiles contained a gene expression signature related to responses to type I interferons (IFN-α/β), MHC class I antigen presentation, activation of tumor necrosis factor receptor subunits and ubiquitination. Conversely, biological processes regulated by the transcripts obtained from T1D subjects *vs*. β-cells exposed to IL-1β + IFNγ, included gene signatures related to immune infiltration like cell adhesion, immunoregulatory interaction between lymphoid and non-lymphoid cells, and PD-1 signaling. The authors also identified several classes of compounds with some potential to revert β-cell inflammation during the natural history of the disease, including bile acids, bromodomain inhibitors, leucine-rich repeat kinase (LRRK) inhibitors and vitamin D receptor agonists. Likewise, Yip and colleagues identified various immune pathways related to cell adhesion, insulin secretion, glucose metabolism and pancreas development that were differentially expressed in the pancreas of autoantibody positive (AAb+) individuals and partially overlapped with changes observed in pre-diabetic NOD mice. The genes included *RGS16, CLEC4D*, and *FCGR2B*, which are enriched in leukocytes and exhibited reduced expression in samples from AAb+ individuals. The authors hypothesized that reduced expression of *FCGR2B* in pre-diabetic individuals, detected in blood and pancreas, could lead to hyper-responsiveness, proliferation and maturation of autoreactive B cells contributing to a loss of tolerance and progression of disease.

PD-1 signaling represents an important negative regulator of T cell activation, with implications for controlling autoreactivity in T1D. Falcone and Fousteri reviewed the role of the PD-1/PD-L1 axis in the maintenance of immunological tolerance, described mechanisms by which this pathway is regulated, and discussed how alterations in this checkpoint could contribute to islet autoimmunity. The authors further discussed how the microbiota may alter the PD-1/PD-L1 axis, as well as recent findings in subjects treated with immune checkpoint inhibitors for cancer immunotherapy (i.e., anti-PD-1 monoclonal antibody). The authors concluded by listing strategies to target this pathway to bolster regulation and avert islet autoimmunity.

Given the interactions between islets and tissue-resident immune cells, multiple authors centered their work on the cross-talk between β-cells and innate immune subsets. Macrophages, dendritic cells (DCs), and neutrophils are often the first cells to interact with potentially abnormal β-cells and provide a link with the adaptive immune system, as suggested in a perspective article by Zirpel and Roep. They highlighted the possible roles of macrophages and DCs, and the importance of further understanding the changes between benign leukocyte residency and pathogenic infiltration. Citro et al. focused on macrophages and neutrophils, and how cytokines in the islet niche could modulate insulin secretion and β-cell function. The authors discussed the importance of their modulation for the protection and/or improvement of islet function. Xing et al. investigated the transcriptional profile of patients with latent autoimmune diabetes in adults (LADA) and identified neutrophilic dysfunction, with enhanced activation of degranulation, adhesion and migration at the transcriptional level. Parv et al. investigated the different polarization of macrophages in the pancreas using a mouse model. The authors found that endocrine-resident macrophages were more efficient at performing efferocytosis, a homeostatic process that clears endogenous cells, and phagocytosis, both *in vitro* and *in vivo*, than exocrine-resident macrophages. The authors point out the need to further understand the intrinsic differences between endocrine and exocrine innate immune cells, with respect to their ability to regulate autoimmunity, providing a potentially interesting new line of investigation.

In addition to innate subsets, B cells within the adaptive arm of the immune system are thought to participate in T1D progression. This notion emanates from their appearance as a prognostic indicator of disease and invites the hypothesis of a role as non-professional antigen presenting cells. However, the exact role of B cells in T1D pathogenesis, particularly within the islet microenvironment, remains poorly understood. Leete and Morgan discuss the preferential localization of B cells in T1D in regard to their detection in peripheral blood, secondary lymphoid organs, and islets. They highlight studies reporting a notable increase in islet B cells in individuals diagnosed with T1D in early life. The authors propose that B cells might promote the activation of autoreactive CD8^+^ T cells in the islets and highlight therapeutic strategies for attenuating B cell function.

Autoreactive T and B cells are linked, and their collaboration can drive immune-mediated pathology. CD4^+^ T cells have been detected at low frequencies in the islets of T1D subjects but their numbers may be more prominent at the earliest stages of the disease. Landry et al. evaluated preproinsulin reactivity among CD4^+^ T cells isolated from islets of six organ donors with T1D. The authors identified 14 T cell receptor (TCR) clonotypes, which recognized proinsulin peptides (A- and B-chain, and C-peptide) presented by various HLA Class II molecules, and observed a trend towards dominant restriction by HLA-DQ. However, citrullination of insulin B-chain peptides did not induce stronger responses in the TCRs reactive against the native form. CD8^+^ T cells reactive against preproinsulin have similarly been found in the pancreas of T1D subjects, and in this Research Topic, Bender et al. review the differences in autoreactive CD8^+^ T cell frequency between peripheral blood and pancreas in individuals with and without T1D, and how cytokines secreted by stressed β-cells could attract CD8^+^ T cells to the islets. The authors highlight the role of the chemokine CXCL10, among other chemokines, for its capacity to attract T cells, which express the CXCL10 receptor, CXCR3. They also discussed numerous studies that have investigated CXCL10 expression in α- and β-cells, with mixed results. In an attempt to provide a definitive answer, Nigi et al. investigated the expression of CXCL10 among endocrine cell subtypes in NOD mice and human tissue. CXCL10 was observed in murine α- and β-cells, but the colocalization and expression increased in α-cells of diabetic mice. Likewise, CXCL10 was preferentially expressed in α-cells in the islets of subjects with T1D while it was absent in control donors. Christen and Kimmel reviewed the role of chemokines in T1D, focusing especially on the CXCL10/CXCR3 axis. They discussed the therapeutic potential of neutralizing chemokines as part of combination therapies with T-cell targeting drugs like anti-CD3. The authors ultimately hypothesize that the blockade of CXCR3 chemotaxis may provide a novel combinatorial strategy to prevent the migration of diabetogenic T cells from secondary lymphatics.

The enrichment of autoreactive T cells within the islets in T1D provides strong evidence for a loss of immune tolerance. Regulatory T cells (Tregs) are found in very low numbers within the islets, and their stability and function are not well understood in the context of human T1D. In their review article, Scherm and Daniel explore the regulatory potential of miRNAs, including how miRNAs regulate effector T cell function, and their use as biomarkers of islet autoreactivity, with a special section dedicated to miRNA regulation of Tregs. In addition to providing a comprehensive list of miRNAs involved in immune regulation, the authors highlight three miRNAs (miR92a-3p, miR181a-5p, miR142-3p) and describe their function in detail.

The articles noted above underscore the critical cellular interactions linking the immune effectors and targets of autoimmunity in T1D. A number of articles highlighted exciting opportunities to direct therapies not only at the immune system, but also to protect β-cells within islets. Indeed, Perna-Barrul et al. discuss the potentially protective role for betamethasone, a drug routinely administered to mothers at risk of preterm birth, on preserving β-cells function and restoring proper interactions with the immune system in early life. The authors speculate that betamethasone could be used as a protective agent shortly before birth or in the perinatal period based on its capacity to make β-cells less immunogenic. Bogdani et al. explored the therapeutic potential of a Vβ13a TCR monoclonal antibody, 17D5. They demonstrate that 17D5 delays spontaneous diabetes onset in DRLyp/Lyp rats. Some rats did not develop disease and retained a high proportion of insulin containing islets, with reductions in hyaluronan deposits, CD68^+^, CD3^+^ and CD8^+^ infiltration.

Overall, the studies outlined in this Research Topic highlight the critical need for a deeper understanding of this organ-specific autoimmune disease and the exigence for therapeutic modalities that can act at the target organ to reduce cellular stress, immunogenicity, and preserve long-term immune tolerance to pancreatic β-cells in subjects with or at risk for T1D.

## Author Contributions

All the authors have made a substantial, direct and intellectual contribution to the work and approved it for publication.

## Funding

Work in the Brusko lab is funded by NIH (UG3 DK122638, P01 AI042288, U54 AI142766), The Leona M. and Harry B. Helmsley Charitable Trust, and the Network for Pancreatic Organ donors with Diabetes (nPOD; RRID : SCR_014641), a collaborative type 1 diabetes research project supported by JDRF (nPOD: 5-SRA-2018-557-Q-R) and The Leona M. & Harry B. Helmsley Charitable Trust (Grant#2018PG-T1D053, G-2108-04793). The content and views expressed are the responsibility of the authors and do not necessarily reflect the official view of nPOD. Organ Procurement Organizations (OPO) partnering with nPOD to provide research resources are listed at http://www.jdrfnpod.org/for-partners/npod-partners/. Work in the Laboratory of RM is funded by grants from The Leona M. and Harry B. Helmsley Charitable Trust (Helmsley no. 1901-03689), the Fondation pour la Recherche Médicale (EQU20193007831) and the Agence Nationale de la Recherche (ANR-19-CE15-0014-01). TR-C is supported by JDRF (5-CDA-2020-949-A-N). RM and TR-C are funded by the Innovative Medicines Initiative 2 Joint Undertaking under grant agreements 115797 and 945268 (INNODIA and INNODIA HARVEST). These Joint Undertakings receive support from the Union’s Horizon 2020 research and innovation programme, European Federation of Pharmaceutical Industries Associations, JDRF and The Leona M. and Harry B. Helmsley Charitable Trust.

## Conflict of Interest

The authors declare that this editorial was prepared in the absence of any commercial or financial relationships that could be construed as a potential conflict of interest.

## Publisher’s Note

All claims expressed in this article are solely those of the authors and do not necessarily represent those of their affiliated organizations, or those of the publisher, the editors and the reviewers. Any product that may be evaluated in this article, or claim that may be made by its manufacturer, is not guaranteed or endorsed by the publisher.

